# EXPRESSION OF CONCERN: Collaboration of MYC and RUNX2 in lymphoma simulates T‐cell receptor signaling and attenuates p53 pathway activity

**DOI:** 10.1002/jcb.29143

**Published:** 2019-06-30

**Authors:** Jodie Hay, Kathryn Gilroy, Camille Huser, Anna Kilbey, Alma Mcdonald, Amanda MacCallum, Ailsa Holroyd, Ewan Cameron, James C. Neil

**Affiliations:** ^1^ Molecular Oncology Laboratory, Centre for Virus Research, Institute of Infection, Immunity, and Inflammation University of Glasgow Glasgow United Kingdom; ^2^ Paul O’Gorman Leukaemia Research Centre University of Glasgow Glasgow United Kingdom; ^3^ School of Veterinary Medicine University of Glasgow Glasgow United Kingdom

**Keywords:** lymphoma, MYC, p53, RUNX, senescence, SMYD2

## Abstract

MYC and RUNX oncogenes each trigger p53‐mediated failsafe responses when overexpressed in vitro and collaborate with p53 deficiency in vivo. However, together they drive rapid onset lymphoma without mutational loss of p53. This phenomenon was investigated further by transcriptomic analysis of premalignant thymus from RUNX2/MYC transgenic mice. The distinctive contributions of MYC and RUNX to transcriptional control were illustrated by differential enrichment of canonical binding sites and gene ontology analyses. Pathway analysis revealed signatures of MYC, CD3, and CD28 regulation indicative of activation and proliferation, but also strong inhibition of cell death pathways. In silico analysis of discordantly expressed genes revealed *Tnfsrf8/CD30*, *Cish*, and *Il13* among relevant targets for sustained proliferation and survival. Although *TP53* mRNA and protein levels were upregulated, its downstream targets in growth suppression and apoptosis were largely unperturbed. Analysis of genes encoding p53 posttranslational modifiers showed significant upregulation of three genes, *Smyd2*, *Set*, and *Prmt5*. Overexpression of SMYD2 was validated in vivo but the functional analysis was constrained by in vitro loss of p53 in RUNX2/MYC lymphoma cell lines. However, an early role is suggested by the ability of SMYD2 to block senescence‐like growth arrest induced by RUNX overexpression in primary fibroblasts.

## Graphical abstract

In this paper, we use transcriptomic analysis to investigate the mechanisms by which MYC and RUNX oncogenes cooperate to drive rapid lymphomagenesis without mutational loss of p53. We show that this gene combination activates pathways to simulate the successful transit of cells through T‐cell repertoire selection. We also show that RUNX2 and MYC collaborate to upregulate the methyltransferase SMYD2, shown by the presence of anchored RUNX/MYC sites, thus functionally inactivating p53 at the posttranslational level.

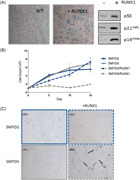

## 1 INTRODUCTION

The oncogenic potential of RUNX2 was first discovered through its identification as a target for transcriptional activation in a retroviral mutagenesis screen in transgenic mice overexpressing MYC in the T‐cell compartment (CD2‐*MYC*).^1^ It was subsequently shown that any of the three murine *Runx* family genes can be selected for activation in this transgenic model,^2^, ^3^, ^4^ suggesting a redundant oncogenic role for RUNX overexpression in the context of MYC‐induced lymphoma. Consistent with this hypothesis, transgenic mice over‐expressing MYC along with either RUNX1 or RUNX2 display rapid onset of T or B‐cell lymphomas.^5^, ^6^ Furthermore, retroviral mutagenesis screens in CD2‐*RUNX2* mice identified both *MYC* and *MYCN* as preferred targets for activation, indicating a strong selection for co‐activation of both gene families as drivers of lymphoma.^7^

The *Runx* genes have also been observed as preferred targets for retroviral activation in Eμ‐Myc transgenic models and in mice deficient in p53 or p19^Arf/Cdkn2a^,^8^ but are rarely seen in end‐stage tumors of wild‐type mice. A rationale for this selective targeting is that the *Runx* genes operate as “conditional” oncogenes, inducing growth arrest when activated in primary cells but driving tumor development when combined with MYC overexpression or loss of function of the p53 pathway.^9^ In support of this hypothesis, overexpression of RUNX2 alone is growth suppressive in early T‐cell development, blocking differentiation and proliferation at the β‐selection stage, but confers predisposition to lymphoma and collaborates strongly with germ‐line inactivation of p53.^7^, ^10^ Moreover, ectopic expression of any of the RUNX family induces senescence‐like growth arrest (SLGA) in primary mouse or human fibroblasts through a process that depends on the integrity of both the p19^Arf^/p53 and p16^Cdkn2a^/Rb arms of the tumor suppressor response.^11^, ^12^, ^13^, ^14^

The CD2‐*MYC* model also displays the phenomenon of conditional oncogenesis, as these mice have a low incidence of lymphoma development, and mice that remain healthy display no detectable expression of the transgene.^15^ Although the CD2 locus control region (LCR) is active from the common lymphoid precursor stage,^16^ spontaneous tumors in the CD2‐*MYC* model display productive T‐cell receptor (TCR) rearrangement and express CD3.^15^ Moreover, analysis of TCR β‐chain usage in CD2‐*MYC* lymphomas suggests that autoreactive cells may be selected.^17^

In light of the potent effect of p53 loss on both CD2‐*RUNX2* and CD2‐*MYC* lymphoma development, it was surprising that the combination of both transgenes led to the rapid development of tumors in which the p53 pathway appears to be intact.^18^ In support of this interpretation, the wild‐type p53 allele is retained in primary tumors in CD2‐*RUNX2*/CD2‐*MYC*/p53^+/−^ mice, but rapidly lost on in vitro culture of lymphoma cell lines which also display de novo activation of the p53‐repressed target p19^Arf^.^18^ Moreover, unlike spontaneous tumors in CD2‐*MYC* mice, CD2‐*RUNX2*/CD2‐*MYC* early onset lymphoma cells display a low apoptotic index along with immunoblastic morphology, indicating that this potent oncogene combination overcomes the propensity of RUNX2 and MYC to induce, respectively, growth arrest and apoptosis.^18^

The molecular mechanism of p53 “bypass” in this context remains unexplained but is addressed in this study where the combinatorial effect of MYC and RUNX2 was examined by transcriptome analysis of thymus tissues from 10‐day old CD2‐*MYC*/CD2‐*RUNX2* mice, in which previous studies have shown a large polyclonal expansion of premalignant cells.^2^, ^6^, ^18^, ^19^ The combination of MYC and RUNX2 orchestrates TCR downstream responses in favor of survival and proliferation. Moreover, our findings indicate that p53 is upregulated but functionally quiescent in prelymphoma cells, suggesting that posttranslational control of the p53 activity is important for potent MYC/RUNX oncogenic synergy.

## 2 METHODS

### 2.1 Cells, constructs, and retroviral transductions

Animals were routinely monitored and killed when showing signs of ill health in line with the UK Animals (Scientific Procedures) Act, 1986. CD2‐*MYC*, CD2‐*RUNX2*, CD2‐*MYC*/CD2‐*RUNX2*, and Runx2/MYC/p53^+/−^ transgenic animals and their maintenance were described previously.^19^ Littermate‐matched genotype controls were used to control for mouse strain. The GIMP cell lines were established by culturing tumor cells arising from Runx2/MYC/p53^+/−^ in vitro. Retroviral transductions of primary murine embryo fibroblasts (MEFs) were performed as described previously.^12^ The retroviral vectors were based on the pBabe plasmid carrying the puromycin‐selectable marker. cDNA encompassing the complete coding sequence of SMYD2 and SMYD5 was cloned into a GFP‐selectable MIGR1 expression vector and used to transfect GP + E86 producer lines. Viral supernatents were then collected and used to transduce primary MEFs before sorting cells expressing GFP. RUNX1‐GFP transductions were as previously described.^5^

### 2.2 Microarray analysis

RNA was isolated and purified from the thymuses of 10‐day‐old wild‐type and CD2‐*MYC/Runx2* transgenic mice as previously described.^2^ Microarray analysis was based on a previously archived dataset (GEO accession number GSE80254). Briefly, whole genome expression profiling was performed using Affymetrix mouse GeneChip microarrays (MoGene‐1) in triplicate as per the manufacturer's protocol (Affymetrix, UK). Data analysis was carried out using Partek Genomics Suite (Partek Inc, St. Louis, MO). After Robust Multichip Average normalization^20^ with GC content pre‐background adjustment, differential expression analysis was performed using ANOVA. Multiple testing correction was performed and “q‐value” cut‐offs selected^21^ with gene changes of q < 0.05 considered significant. Graphical representations of data were prepared using CLC Genomics Workbench 4.^2^

### 2.3 Cell proliferation and senescence staining

The CellTiter‐Glo Luminescent Cell Viability Assay was performed as per the manufacturer's instructions. Briefly, the CellTiter‐Glo assay reagents were prepared according to the manufacturer's protocol. Next, in a white 96‐well plate (Nunc, Thermo Fischer Scientific), 25 µL of cell suspension was added along with 25 µL of prepared CellTiter‐Glo reagent. Plates were then incubated for 30 minutes at room temperature before luciferase activity was determined using a luminometer. Growth curves were performed over 15 days in triplicate using trypan blue as a vital stain, whereas senescence‐associated beta galactosidase (SA‐β‐gal) staining was assayed in parallel after 8 days, both as described previously.^12^

### 2.4 Western blots and antibodies

Preparation of protein extracts from whole cells or day 10 mouse thymus tissue was performed as described previously.^12^ Samples equivalent to 30 μg total protein (Bio‐Rad protein assay) were resolved on 8%, 10%, or 17% SDS‐polyacrylamide gels and transferred to enhanced chemiluminescence (ECL; Amersham) nitrocellulose membranes. The antibodies used were α‐p16^INK4a^, α‐p21^WAF1^, α‐actin (sc‐1207, sc‐471, and sc‐1616, respectively; Santa Cruz Biotechnology), α‐p19^ARF^ (ab80; Abcam), α‐p53 and α‐SMYD2 (IC12 and D14H7, respectively; Cell Signaling Technology). Western blots were developed using ECL reagent according to the manufacturer's protocol.

### 2.5 Bioinformatic analyses

Microarray analysis was performed and significantly changed genes were defined as described above. Principal component analysis (PCA) was performed in R using the base statistics functions then visualized using the “Rgl” package. Transcription factor binding site enrichment analysis for various gene subsets was performed using the “Anchored Combination Site Analysis tool” from the oPOSSUM 3.0 suite of tools for anchored RUNX2/MYC binding (with MYC as the anchoring site and RUNX as the anchored site), or the “Single Site Analysis” tool for MYC or RUNX2 sites individually, using default settings.^22^ Gene Ontology analysis using DAVID was performed for the stated gene lists using default settings. The Ingenuity Pathway Analysis platform (IPA, Qiagen) was used to perform pathway analysis by loading gene IDs, fold‐changes, and q‐values from the stated gene lists, then performing a core expression analysis using default settings. Heatmaps, barcharts, Venn diagrams, and scatterplots were produced using custom R scripts.

## 3 RESULTS

### 3.1 Premalignant thymus reveals a co‐operative program of transcriptional regulation by MYC and RUNX2

As reported previously and illustrated in Figure 1A, CD2‐*MYC/*CD2‐*Runx2* mice develop rapid onset thymic lymphoma with complete penetrance whereas the parental CD2‐*Runx2* and CD2‐*MYC* strains display low lifetime rates of tumor development.^6^ To explore further the mechanism of this potent oncogene collaboration, we analyzed early transcriptome changes in the thymus of 10‐day‐old CD2‐*Runx2*/CD2‐*MYC* mice. These mice harbor a large premalignant population comprising a thymocyte fraction that is 40%‐60% polyclonal, as assessed by rearrangement of TCR β‐chain genes. This premalignant phase precedes the development of oligoclonal end‐stage thymic lymphomas that disseminate to peripheral lymphoid tissues and other organs at age 4 to 6 weeks.^2^, ^6^, ^19^ RNA samples extracted from 10‐day thymus tissues were analyzed by Affymetrix gene expression microarray (MoGene‐1) and compared with normal controls from the same strain background (C57/CBA).^2^ Parental CD2‐*MYC* and CD2‐*Runx2* strains were not analyzed here as most 10‐day‐old CD2‐*MYC* mice display no detectable transgene expression in thymus,^15^ whereas CD2‐*Runx2* mice at the same stage display only a small abnormal population of CD8 ISP cells with low proliferative capacity.^10^ The transcriptomic dataset was deposited previously during our study of lymphoma progression and retroviral mutagenesis^2^ but is used here for the first time to address the mechanistic basis of the MYC/RUNX2 oncogene collaboration.

**Figure 1:**
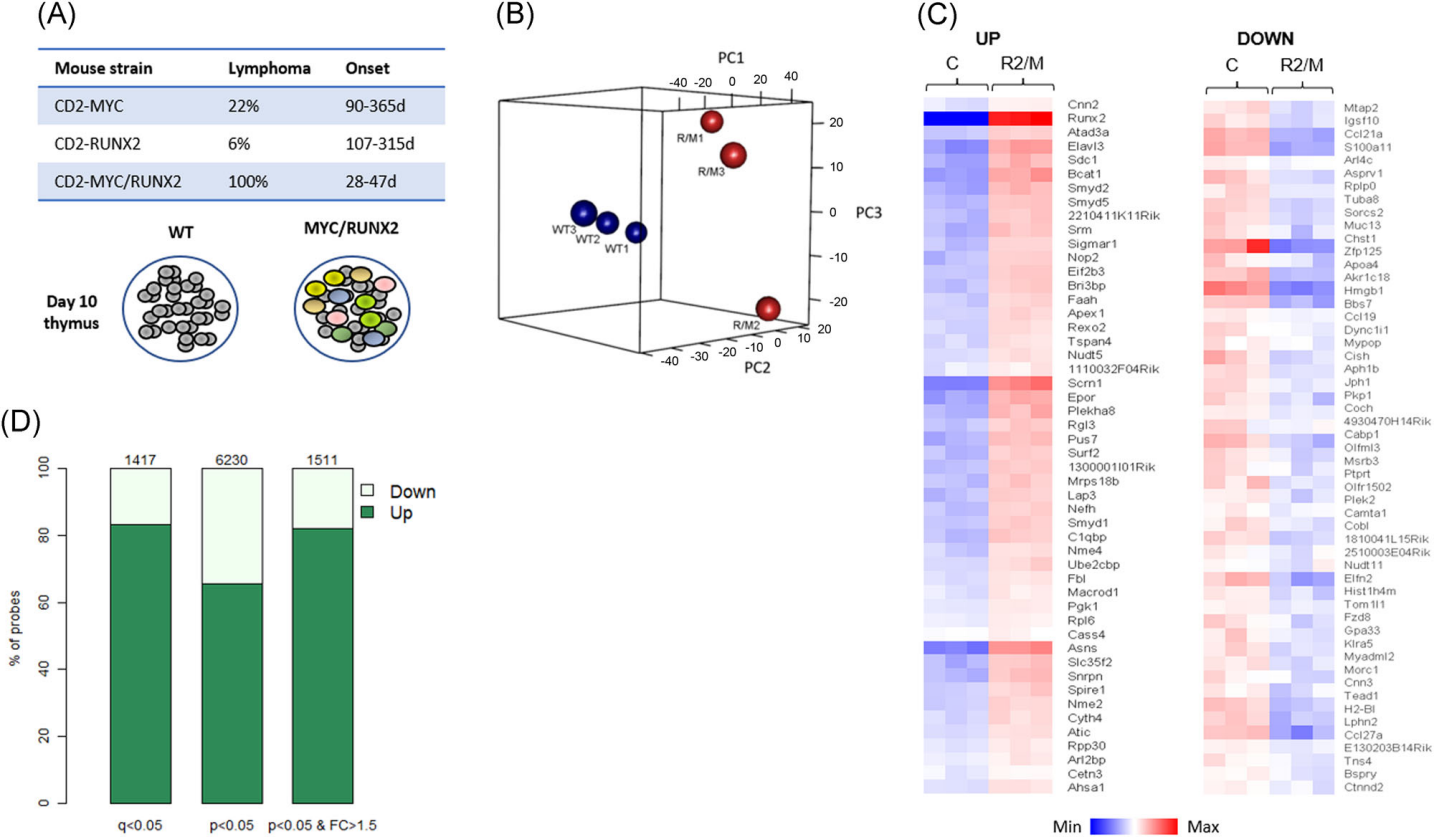
Transcriptome analysis of premalignant thymus in CD2‐*RUNX2*/CD2‐*MYC* mice. A, Summary of key features of the transgenic mouse models, as previously published.^6^, ^18^ Top panel: Table showing the relative frequency and time of onset of T‐cell lymphoma in CD2‐*RUNX2*/CD2‐*MYC* and parental mouse strains; bottom panel: schematic of 10‐day‐old RUNX2/MYC transgenic thymus showing polyclonal immunoblasts (colored) alongside phenotypically normal lymphocytes (gray).^18^ B, Principal Component Analysis (PCA) plot of microarray expression data, comparing 10‐day‐old thymus from WT and RUNX2/MYC mice (blue and red spheres, respectively). C, Heatmaps showing the top 50 up‐ and downregulated genes ranked by significance. Shown are replicates for WT control and transgenic RUNX2/MYC samples (C and R2/M, respectively). D, Bar plot showing the proportion of probes that are up‐ or downregulated using the significance thresholds shown. The numbers above the bars indicate the total number of significant changes for each threshold

Principal Component Analysis demonstrated good separation of the WT and RUNX2/MYC sample groups, and showed a higher divergence between the transgenic samples, indicative of increased polyclonality (Figure 1B). The most significantly changed genes are illustrated in the heat map shown in Figure 1C. As shown in Figure 1D, a q = < 0.05 cut‐off revealed a preponderance of upregulated genes, with almost five times as many significantly upregulated as downregulated genes (896 and 171 genes, respectively, from 1178 and 239 individual up and downregulated probes, respectively; Table S1). This bias towards upregulation was not observed in the much larger dataset generated by a *P* = < .05 cut‐off, but was restored by adding a filter for probe sets changed by >1.5‐fold. This indicates that the major difference is in the magnitude of changes in the upregulated set, and was observed previously for MYC‐regulated genes during the controversy over whether MYC acts as a “universal amplifier” that upregulates virtually all expressed genes^23^ or as an on‐off regulator of key target genes in cancer cells.^24^, ^25^

To explore the roles of MYC and RUNX2 in regulation, the genes meeting the q‐value significance threshold were analyzed for MYC and RUNX consensus binding motifs using a public domain toolset (http://opossum.cisreg.ca/oPOSSUM3/; Figure 2A). This analysis revealed a highly significant enrichment of canonical MYC sites within 1 kb upstream and 500 bp downstream of the promoters of upregulated genes, but not in the downregulated set. Similar enrichment was also noted at up to 10 kb from the target promoters, consistent with evidence that overexpressed MYC can regulate by enhancer “invasion” as well as by direct action at the promoter.^23^ In contrast, RUNX motifs were enriched near the promoters of the downregulated set and distal to promoters of the upregulated set. Intriguingly, anchored combination site analysis revealed enrichment of closely linked MYC and RUNX sites near the promoters of a subset of both up‐ and downregulated gene sets. Taken together, these observations suggest a dual role for MYC and RUNX in the regulation of both independent and overlapping gene sets, with potential direct coregulation of a critical subset. Although this analysis would not detect long‐range *cis* interactions or non‐DNA binding interactions, it indicates that direct DNA binding interactions play a significant role in their oncogenic collaboration. The genes and locations of relevant motifs scored in the anchored transcription factor site analysis is presented in Table S2.

**Figure 2:**
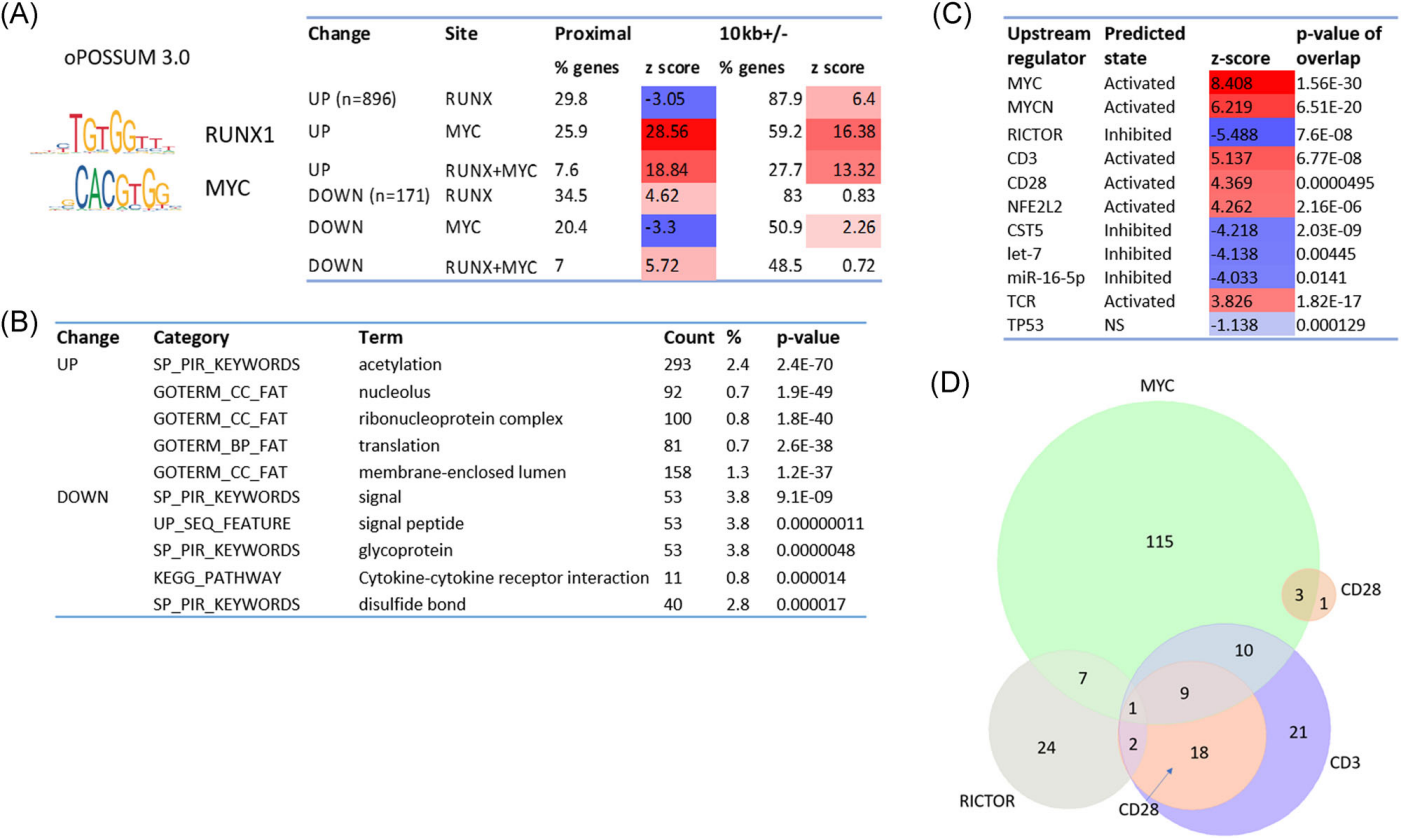
The functional analysis of transcriptomic changes in pre‐lymphomatous mouse thymus. A, The oPOSSUM analysis of RUNX and MYC binding sites. Left panel: Consensus binding sites for RUNX and MYC used in the analysis. Right panel: Table showing the percentage of RUNX, MYC, or RUNX + MYC binding sites found adjacent to the up‐ or downregulated genes (Proximal, defined as 1 kb upstream and 500 bp downstream of the promoter) or in the upstream promoter region (defined as up to 10 kb upstream or downstream of the promoter region). Significance is indicated with Z‐score, with those in red showing enrichment and those in blue showing fewer sites than expected. B, DAVID Gene Ontology analysis of the genes that are up‐ and downregulated in control vs RUNX2/MYC transgenic mouse thymus, showing the functional annotations that are most enriched. C, The top predicted upstream regulators of genes significantly changed in 10‐day RUNX2/MYC transgenic thymus (Ingenuity IPA). Significance is indicated using Z‐score. Predicted activation or inhibition states (red or blue bars, respectively) are as indicated. D, Weighted Venn diagram showing the overlap of the gene sets regulated by the top upstream regulators noted in panel C

As shown in Figure 2B, analysis by the DAVID functional annotation tool^26^ (https://david.ncifcrf.gov/home.jsp) also revealed a striking difference between the up‐ and downregulated genes, as only the former displayed highly significant enrichment for cell growth and metabolic processes typically associated with MYC overexpression.^27^ The downregulated set revealed much less potent enrichment and favored gene ontology terms indicative of cell surface and secreted components. Intriguingly, this enrichment of surface and signaling molecules resembles the pattern we previously observed in fibroblasts over‐expressing *Runx* genes, albeit with little overlap between the target gene lists.^28^ These observations mirror the binding site enrichment data and suggest that MYC drives a major component of the upregulated gene expression, whereas a subset of activated and repressed genes may be subject to dual control.

### 3.2 Perturbation of T‐cell signaling in premalignant MYC/RUNX2 thymus

We next analyzed the altered gene set and their associated fold‐changes in expression using Ingenuity Pathway Analysis. Predicted upstream regulators of the altered gene sets provide the strongest scores for MYC and the related MYCN, both by *P* value of overlap and the activation z‐score. Figure 2C lists the top ten upstream regulators, ranked by activation score, as well as TP53 for comparison. A strong bias towards T‐cell signaling pathways was evident from the positive activation state of TCR, CD3, and CD28 coreceptor pathways, whereas the most potently inhibited regulator was RICTOR (a component of mTORC2). Although there was strong overlap between the gene sets denoting CD3 and CD28 activation, these appeared largely distinct from MYC activation and RICTOR inhibition, suggesting that these are functionally distinct aspects of the MYC/RUNX2 oncogenic program (Figure 2D). Downregulation of RICTOR‐dependent processes appears somewhat counter‐intuitive in light of the association of RICTOR with growth signaling. However, loss of RICTOR has been reported to remove a block to T‐cell proliferation because of limiting arginine and leucine levels,^29^ suggesting that MYC/RUNX2 collaboration may also entail loss of this critical metabolic checkpoint. Strongly inhibitory states were also inferred for CST5 (cystatin D) and the growth inhibitory microRNAs let‐7 and miR‐16‐5p. These upstream regulators each have functional links to p53.^30^, ^31^, ^32^ However, despite a strong *P* value overlap indicating p53 as an upstream regulator of the altered gene set, the activation state of p53 itself was ambiguous with a majority of targets indicating inhibition but a substantial minority denoting activation (Figure 2C).

The evidence for activation of pathways downstream of TCR/CD3 is interesting as although RUNX2/MYC 10‐day thymus shows marked upregulation of surface TCRαβ and CD3, the aberrant population of CD8+ blast cells also occurs in TCRα deficient mice, arguing that this expansion is independent of TCR ligation.^18^ Moreover, stimulation of immature thymocytes through CD3 or activation of MYC normally leads to death by apoptosis as a sequel to activation and proliferation,^33^, ^34^ whereas RUNX2/MYC thymocytes proliferate with a remarkably low apoptotic index.^18^ These observations imply that the normal self‐limiting processes that follow CD3 stimulation or MYC activation are not functional in these cells. For clues to the basis of this phenomenon, we focused on the small set of target genes that were regulated in the opposite manner from expected in response to TCR/CD3 and MYC by pathway analysis (highlighted in yellow in Table S3).

As shown in Table 1, a total of 24 unique genes were regulated in a manner discordant with the overall prediction of pathway activation for the top upstream regulators MYC, TCR, CD3, and CD28. These genes were also significantly enriched for MYC and RUNX binding motifs and for closely linked sites both proximal and distal to the promoter regions. Several genes were discordant for multiple regulators, including *Tnfrsf8/CD30*, *Ly6a/Sca1*, *Il13*, and *Cish*. These are strong candidates for dual regulation by MYC and RUNX2 to abolish normal regulatory constraints on activation and proliferation.

**Table 1:** Discordantly regulated gene targets of top upstream regulators

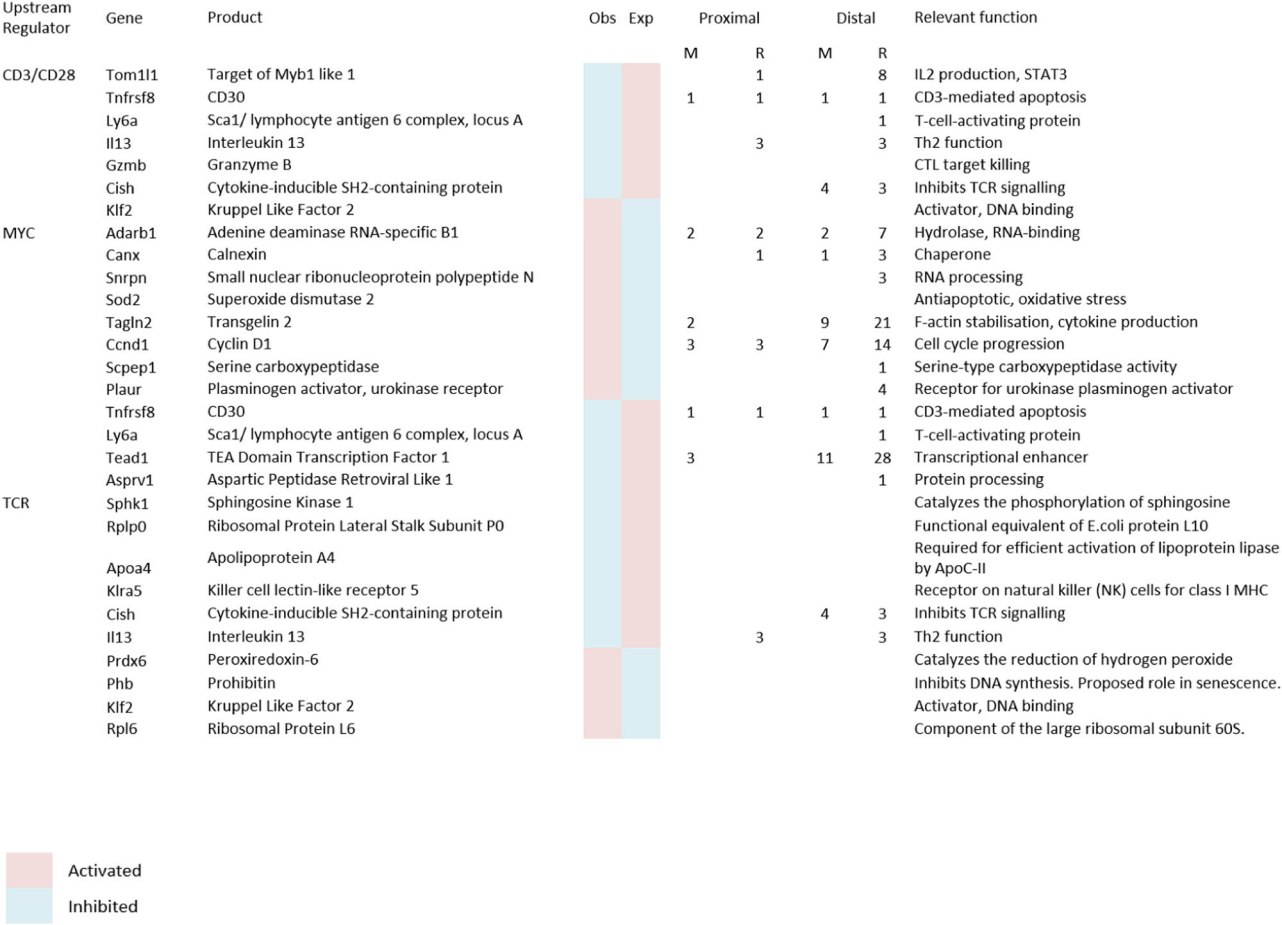

Details of the genes discordantly regulated by the stated upstream regulators, as determined by the Ingenuity IPA analysis and noted in Table S3. For each gene, the observed and expected state is shown to be either activated (red) or inhibited (blue). The number of Runx (R) or Myc (M) binding sites located proximal or distal to the promoter region, as stated, determined by oPOSSUM‐3 analysis, are shown for each gene. The absence of a numeral indicates no sites were found. The full names and relevant functions of the genes are shown.

### 3.3 Analysis of p53 and target genes in RUNX2/MYC thymus provides evidence of functional attenuation

The ambiguous activation state of p53 in RUNX2/MYC thymus is graphically illustrated by the IPA mechanistic network function (Figure 3A), which showed discrepancies with regard to the expected response of TP53 to MYC activation and expression of the p53 downstream target CDKN2A (yellow lines). Discordant observations were also evident for cytokine signaling through STAT5A. Notably, p53 itself was observed to be overexpressed at the transcriptional level whereas most of its activation targets were expressed at similar levels to control, as shown in the scatterplot in Figure 3B. These observations rule out simple transcriptional repression of p53 as the basis for its aberrant functional readouts.

**Figure 3:**
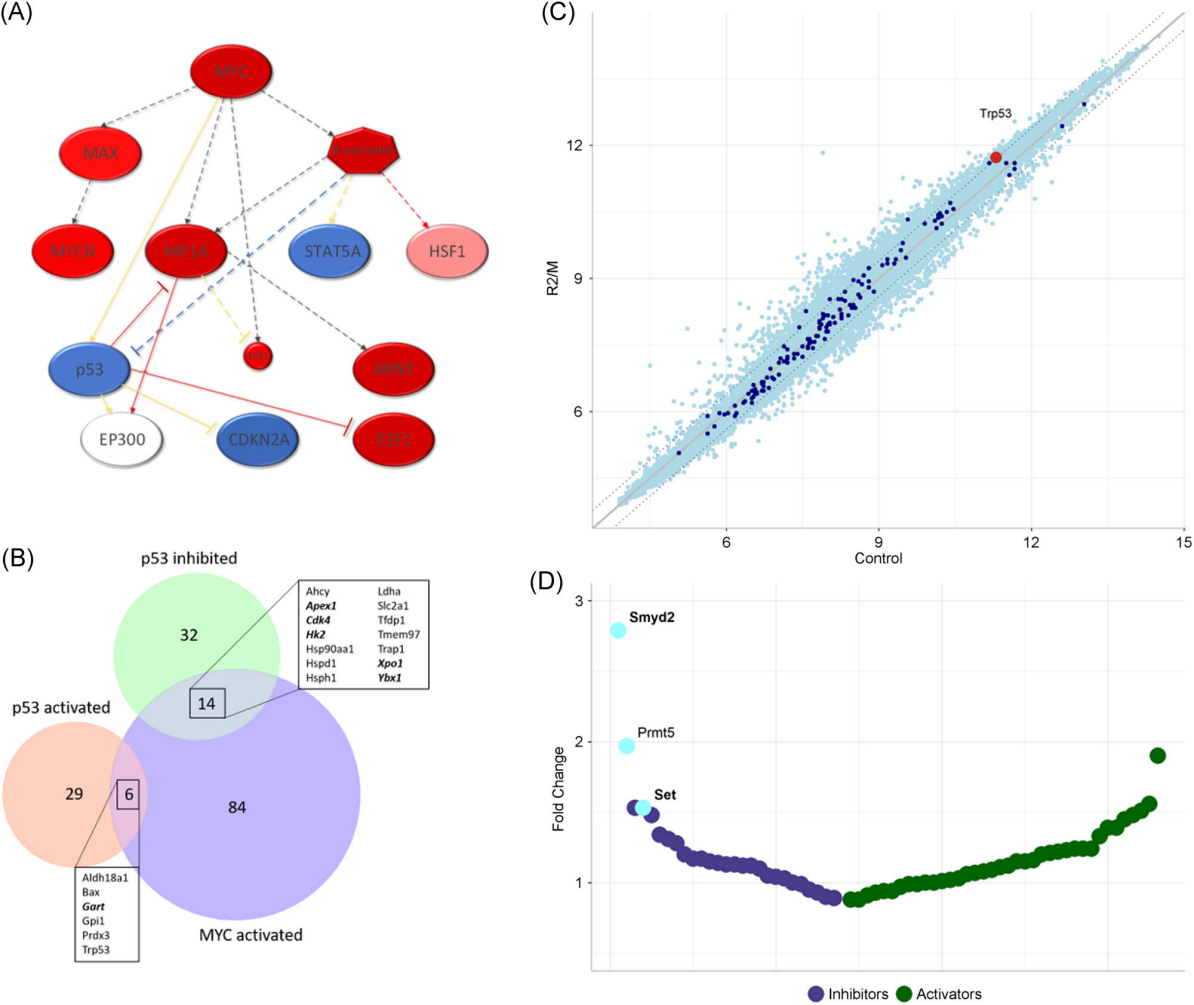
Pathways altered in RUNX2/MYC transgenic thymus. A, Network diagram showing MYC‐regulated pathways altered in RUNX2/MYC thymus, adapted from Ingenuity IPA. Shown are genes with predicted activation (red) or inhibition (blue), with darker colors indicating higher confidence. Red arrow: predicted to lead to activation; blue arrow: predicted to lead to inhibition; gray arrows: no prediction; yellow arrow: the measured findings are inconsistent with the activation state of the downstream molecule. B, Venn diagram showing the interaction between gene sets with either MYC as an upstream regulator (activating) or p53 as an upstream regulator (activating or inhibiting). Bold italics denotes genes with anchored RUNX/MYC binding sites. C, Scatterplot showing the expression of all genes (light blue) and known p53 targets (dark blue)^35^ in WT and RUNX2/MYC thymus (Control and R2/M, respectively). p53 is highlighted in red. A fold‐change of 1 (no change) is depicted by a solid gray line, whereas fold‐changes of +/− 1.4 are shown as dashed lines. D, Plot showing the fold‐change in expression of p53 modifying enzymes in RUNX2/MYC thymus. Enzymes conferring inhibiting modifications (blue) and activating modifications (green) are shown. The light blue modifiers are those that are significantly altered. Bold indicates the presence of anchored RUNX/MYC binding sites

Inspection of the genes denoting p53 inhibition by IPA showed a total of 46 genes, 41 of which were upregulated in contrast to pathway prediction, whereas five were downregulated (Figure 3C and Table S3). Fourteen of the upregulated genes were also annotated MYC activation targets, in contrast to none of the downregulated genes, suggesting that their overexpression in this context is the result of a dominant MYC effect on transcriptional upregulation (Table S3). The downregulated genes included Ly6a/Sca‐1 and Gzmb, which overlap with the discordant sets for MYC and CD3/TCR. Over‐representation of MYC and RUNX motifs was again evident in the p53 inhibited set, and functionally relevant targets with closely linked sites for MYC and RUNX included Cdk4, Xpo1, and Ybx1 (bold italics in Figure 3C). The full gene set and their relevant functions are detailed in Table S4. The possibility that the overexpressed p53 is controlled at the posttranslational level was also investigated, and all annotated modifiers of p53 were analyzed for transcriptional status in RUNX2/MYC thymus. As shown in outline in Figure 3D and in detail in Table S5, there was a preponderance of upregulation of p53‐modifying enzymes over downregulation, and altered genes included both activators and repressors of p53, showing that competing influences impinge on p53 in the milieu of nascent thymic lymphoma. However, only three genes passed the q = < 0.05 significance bar and all are posttranslational inhibitors of p53. The greatest fold‐change affected Smyd2, which encodes a lysine methyl transferase that suppresses p53 trans‐activation by monomethylation (at residue K370 in human p53).^36^ *SMYD2* has been implicated as a target for copy number gain and overexpression in numerous cancer types^37^ and has been shown to be required for MLL‐AF9 leukemia mouse models where RUNX functions have also been implicated.^38^, ^39^ Moreover, interest in SMYD2 as a potential oncogenic driver has led to the identification of several small molecule inhibitors,^40^, ^41^, ^42^ making this an attractive target for further investigation.

### 3.4 Protein expression analysis of RUNX2/MYC 10‐day thymus validates SMYD2 and p53 target gene expression status

Western blot analysis of cells extracted from 10‐day‐old RUNX2/MYC mouse thymus (n = 3) compared with controls confirmed that SMYD2 is overexpressed at the protein level (Figure 4A). This analysis also confirmed that p53 protein levels were at least as high as controls, although one sample displayed evidence of higher molecular weight forms suggestive of posttranslational modification. Significantly, expression of p21^Waf1^, a major downstream effector of p53 growth arrest, was not elevated, and was lower than two of three control samples. However, expression of p19^Arf^, a target of feedback repression by p53 43 was not observed in thymus. As reported previously^18^ and shown in Figure 4B, high expression of p19^Arf^ in RUNX2/MYC/p53^+/−^ lymphoma lines that rapidly lose the wild‐type allele and p53 protein expression on establishment in vitro argues strongly that aberrant in vivo activity is not because of direct mutation of either p19^Arf^ or p53.

**Figure 4:**
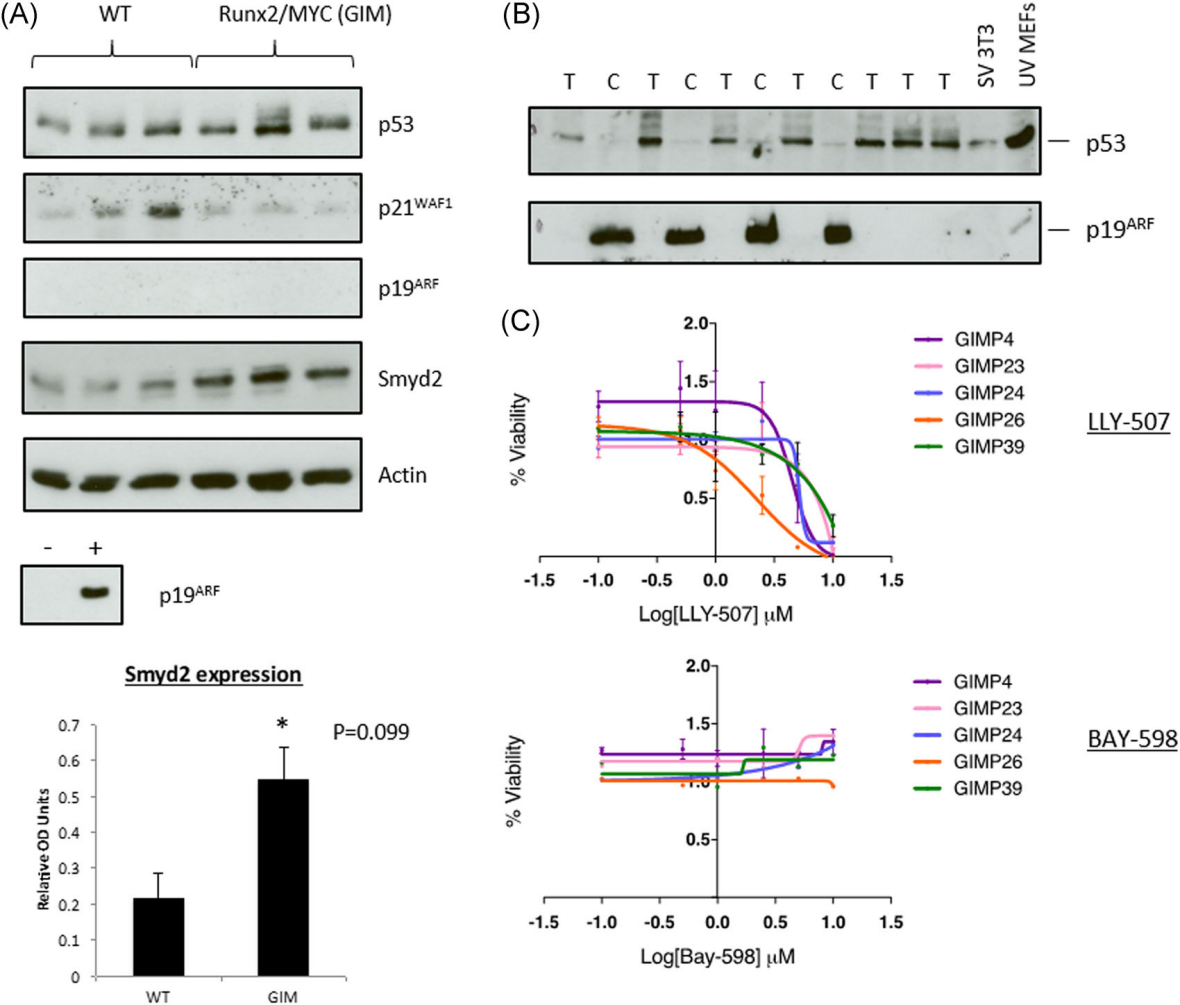
Smyd2 protein expression and inhibition. A, Total protein was extracted from day 10 thymus tissue derived from WT and CD2‐*Runx2*/CD2‐*Myc* (GIM) transgenic mice, as indicated, and equivalent concentrations probed against antibodies to p53, p21^WAF1^, p19^Arf^ and Smyd2 (upper panel). Actin was used as an internal loading control for quantification of Smyd2 (bar chart, lower panel). Positive and negative controls used for p19^Arf^ detection were SV3T3 and NIH3T3 cell extracts, respectively. B, Western Blot analysis as in A using protein extracts prepared from tumors (T) or tumor‐derived cell lines (C) from CD2‐*Runx2*/CD2‐*Myc* animals. Blots were probed against antibodies to p53 and p19^Arf^. C, Metabolic inhibition of cell lines derived from Runx2/Myc/p53^+/−^ mice (GIMP, as shown), as measured using the CellTiter‐Glo assay. Upper and lower panels depict changes in cell viability following treatment with LLY‐507 or BAY‐598, respectively, at the indicated concentrations

### 3.5 RUNX2/MYC/p53^−/−^ lymphoma cell lines are insensitive to small molecule SMYD2 inhibitor BAY‐598

Several specific inhibitors of SMYD2 have been identified by screening small molecule inhibitor libraries. These are chemically diverse but act as substrate competitive inhibitors, inhibiting p53 monomethylation in vitro.^44^ Our initial studies with the lead compound AZ505^45^ showed no discernible activity against RUNX2/MYC lymphoma lines but were not available to us for in vivo use. We therefore tested more potent compounds, LLY‐507^42^ and BAY‐586.^40^ As shown in Figure 4C, only LLY‐507 induced significant metabolic inhibition as measured by CellTiter‐Glo assay, and this was at a relatively high concentration (0.5‐1 μM) compared with the reported IC_50_ for p53 methylation. The other compound, BAY‐598, displayed no obvious toxicity. During our study, a report was published highlighting the off‐target effects of LLY‐507 and the dispensability of both SMYD2 and SMYD3 for the viability and proliferation of a large series of cancer cell lines.^44^ Our findings are in accord with this study but do not exclude a role for SMYD2 in vivo or under stress conditions.^46^

### 3.6 SMYD2 blocks RUNX‐induced SLGA in primary fibroblasts

The oncogenic potential of *Runx* gene overexpression is constrained in normal cells by SLGA, a process that entails upregulation of p19^Arf^/p53 and p16^Cdkn2a^, and requires genetic integrity of both the p53 and Rb pathways.^11^, ^12^, ^13^, ^14^ To explore the potential of SMYD2 to abrogate this response, a cDNA encompassing the complete coding sequence was cloned into the MIGR1 expression vector and transfected into primary MEFs. As all three Runx genes elicit an indistinguishable SLGA phenotype, we analyzed the effects of Smyd2 on Runx1, the most thoroughly analyzed family member.^14^ For comparison, the effects of the methyltransferase Smyd5 were also investigated. As expected, Runx1 induced SLGA in MEFs (Figure 5A). Notably, although Smyd5 had little effect on SLGA in MEFs, Smyd2 expression both rescued the growth arrest induced by Runx1 in MEFs (Figure 5B) and blocked SLGA as indicated by the expression of SA‐β‐gal (Figure 5C), suggesting a specific role for this enzyme.

**Figure 5:**
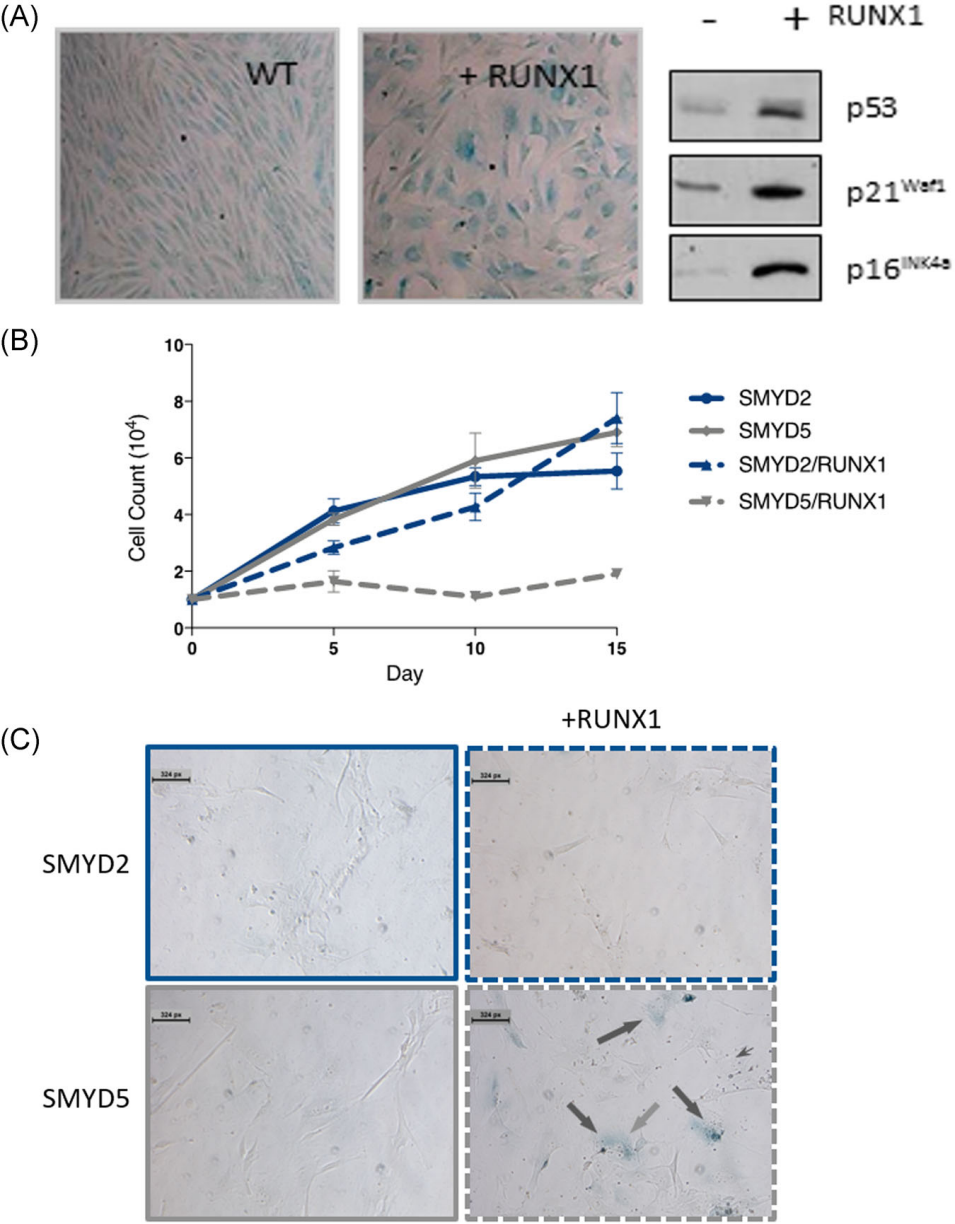
The effect of Smyd2 on Runx1‐induced SLGA. A, Primary MEFs were transduced with either a vector containing *Runx1* or the pBabe‐PURO control, then SA‐β‐gal staining was performed after 8 days in culture (left and center panels). Parallel plates were harvested at the same time and cell extracts analyzed for p53, p21^WAF1^, and p19^Arf^ protein expression (right panel). B, Growth curves for fibroblasts transduced with Smyd2 or Smyd5, with or without Runx1, as indicated. C, SA‐β‐Gal staining of fibroblasts transduced with Smyd2 or Smyd5, with or without Runx1, as indicated. MEFs, murine embryo fibroblasts; SLGA, senescence‐like growth arrest

## 4 DISCUSSION

This study sheds light on the extremely potent synergy of *Runx* and *Myc* family genes in lymphomagenesis and the ability of this oncogene combination to counteract tumor suppressor responses. In silico analysis reveals pathways underlying the aberrant proliferation and survival of nascent lymphoma cells, and identifies genes likely to be involved in the subversion of failsafe processes that precede rapid onset lymphomagenesis.^18^ As MYC and RUNX overexpression have been implicated in a wide range of malignancies, these observations may be of wide relevance to cancer.^9^

The analysis of significant gene expression changes in premalignant RUNX2/MYC thymus revealed a strong preponderance of gene upregulation over downregulation, recapitulating the observations that fueled controversy on the role of Myc as a “universal amplifier” 23 or an on/off switch of gene expression.^24^, ^25^ As the upregulated genes with Myc signatures displayed much greater fold‐changes, it is understandable that the role of Myc, as a negative regulator, is obscured by the application of standard significance thresholds. Although in silico analysis of canonical MYC and RUNX binding sites identifies only a subset of targets, potent enrichment of MYC motifs in the promoters of altered genes indicates that a substantial number are under direct control. As enrichment is also observed up to 10 kb away, it appears that *MYC* expression under CD2 LCR control leads to enhancer “invasion” as reported in other overexpression systems.^24^, ^25^ In contrast, previous transcriptomic analysis of target genes responding to ectopic RUNX1, 2, and 3 expression showed a common program with a balance between positive and negative regulation of target gene expression, reflecting the ability of these regulators to recruit activating or repressive complexes in different chromatin and cellular contexts.^28^, ^47^ Moreover, Runx factors operate on distal enhancer and silencer elements^48^, ^49^ and can regulate gene expression through effects on chromatin structure that do not require stable binding.^50^ In this study the downregulated gene set displayed enrichment of RUNX but not MYC motifs, suggesting that RUNX2 is the principal “off‐switch” for this gene set. Of particular interest was the enrichment of closely linked MYC and RUNX motifs in a subset of both positively and negatively regulated genes, identifying candidate genes for dual control.

This analysis also illuminates earlier observations on the interplay between ectopic MYC and RUNX expression in T‐cell development and the processes of positive and negative selection of T‐cells which are normally spatially and temporally separated in the thymus.^51^ We showed previously that CD2‐RUNX2 transgenic mice display aberrant survival of a small population of immature CD8 ISP cells undergoing β‐selection that would normally be destined for “death by neglect”.^10^ In contrast, MYC overexpression enhances thymic positive selection while also priming cells for apoptosis.^33^ A similar functional relationship has been observed in vitro in T‐cell hybridomas that undergo apoptosis after CD3/TCR activation, a process that requires endogenous MYC/MAX activity 52 but is blocked by ectopic RUNX expression.^53^ We suggest that the potency of MYC/RUNX collaboration in lymphomagenesis reflects the ability of this oncogene combination to simulate successful transit of cells through repertoire selection while blocking the processes that limit unscheduled proliferation. A “simulation” model appears most likely as the early expansion and blast cell phenotype observed in vivo occurs on a TCRα‐deficient background.^18^

In light of these prior observations, the strong IPA prediction of CD3/TCR and CD28 as upstream regulators of the aberrant gene expression program in premalignant RUNX2/MYC thymus in this study sheds light on the likely targets involved. Moreover, a search among discordantly regulated genes downstream of CD3/TCR and MYC for those potentially coregulated by MYC and RUNX revealed a number of targets with the potential to account for the failure of CD3/MYC‐mediated apoptosis, including Tnfrs8/CD30,^54^ Interleukin‐13^55^ and the SOCS family member Cish.^56^ Although the functional analysis is beyond the scope of this study, it would be of great interest to examine the roles of these targets in lymphoma development in vivo.

The expression of *Trp53* mRNA is significantly elevated in RUNX2/MYC thymus, and we noted modest overexpression of p53 protein compared with control thymus from age‐matched mice. The fact that p53 activation targets are largely unaffected despite the expression of two p53 agonists (RUNX2 and MYC) whereas the p53‐p19^Arf^ feedback loop^43^ remains intact argues that the p53 response is controlled at the posttranscriptional level in premalignant cells.

Transcriptome analysis of premalignant RUNX2/MYC thymus for genes encoding known posttranslational modifiers of p53 revealed three inhibitory factors that were significantly upregulated. The most highly upregulated was Smyd2, a gene encoding a lysine methyltransferase that has been associated with poor prognosis in a wide range of leukemias and solid tumors^37^, ^57^, ^58^, ^59^, ^60^ and has been targeted for drug development.^40^, ^41^, ^45^ Our results confirm recent findings that the reported SMYD2 inhibitors behave discordantly because of off‐target effects, and the resistance of RUNX2/MYC cell lines to specific inhibitors recapitulates a wider CRISPR‐Cas study of cancer cell lines.^44^ However, RUNX2/MYC lines show consistent deletion of p53, whereas primary tumors and transplanted lymphomas often retain the wild‐type allele,^18^ arguing that in vivo and in vitro growth selection are markedly different processes. Moreover, as SMYD2 inhibition is synergistic with genotoxic stress^40^, ^46^ it is possible that in tumor cell lines loss of tumor suppressor function and benign culture conditions allow survival without functional SMYD2. RUNX overexpression in primary fibroblasts induces senescence‐like growth arrest in a p53‐ and p16‐dependent manner,^12^, ^13^, ^14^ and our observation that ectopic SMYD2 can rescue cells from this process provides support for a role in cells with intact tumor suppressor pathways. Moreover, the observation that *Smyd2* knockout prevents development of MLL‐AF9 leukemia in a mouse model^38^ reveals an essential role in at least some cancer types, whereas the requirement of MLL leukemias for the RUNX gene activity suggests a functional link to the present findings.^61^, ^62^ Although beyond the scope of this study, in future it will be interesting to test the sensitivity of primary RUNX2/MYC lymphomas to the loss of SMYD2 function in vivo.

## CONFLICT OF INTEREST

The authors declare that there are no conflict of interest.

## AUTHOR CONTRIBUTIONS

JCN, JH, KG, CH, AK, and EC conceived and designed experiments. JH, KG, CH, AK, AMcD, and AMacC performed experiments and JH, KG, CH, and AK analyzed data. JCN wrote the manuscript, which was edited by JH, KG, and AK. AH helped with manuscript preparation.

## DATA AVAILABILITY

Microarray data is publically available and archived (GEO accession number GSE80254).

## Supporting information

Supporting information

Supporting information

Supporting information

Supporting information

Supporting information
